# Traumatic Floating Clavicle: A Case Report and Updated Literature Review

**DOI:** 10.7759/cureus.67630

**Published:** 2024-08-23

**Authors:** Chittawee Jiamton, Pariwat Taweekitikul, Techit Leelasestaporn, Pongsakorn Rungchamrussopa, Thongchai Laohathaimongkol

**Affiliations:** 1 Department of Orthopedics, Queen Savang Vadhana Memorial Hospital, Chonburi, THA; 2 Department of Orthopedic Surgery, Institute of Orthopedics, Lerdsin Hospital, Bangkok, THA

**Keywords:** surgical management, pan-clavicular dislocation, bipolar injury, bipolar dislocation, floating clavicle

## Abstract

Traumatic floating clavicle or bipolar dislocation is a rare injury. Herein, we present a case of ipsilateral sternoclavicular and acromioclavicular joint dislocation after a motorcycle accident. The patient was a 43-year-old man who presented with right shoulder pain and limited range of motion. The radiograph revealed superior displacement of the acromioclavicular joint dislocation and suspected ipsilateral sternoclavicular joint dislocation and a CT scan confirmed injuries to both the medial and lateral ends of the clavicle. Due to the patient being active and young, we considered operative treatment. The sternoclavicular joint was stabilized with FiberTape® suture (Arthrex, Naples, FL), and the acromioclavicular joint with Dog Bone™ Button (Arthrex) and suture cerclage. At the one-year follow-up, the patient reported excellent outcomes without complications. We also summarize the literature on this particular injury, including its characteristics, surgical options, and treatment outcomes.

## Introduction

The "traumatic floating clavicle" [1-5], “bipolar dislocation” [1,6,7], or “pan-clavicular dislocation” [8] injury is a rare and complex condition characterized by simultaneous dislocation of the clavicle in both medial and lateral ends. Additionally, there are reported variations of this injury, known as “bipolar injury” [9], which include fractures at either end of the clavicle. This unique injury pattern results in a discontinuity of the shoulder girdle, leading to significant instability and functional impairment. Floating clavicle injuries are typically caused by high-energy trauma such as motor vehicle accidents, falls from significant heights, or direct blows to the shoulder region. The incidence of this injury is relatively low, accounting for a small percentage of shoulder girdle injuries, but it poses considerable challenges in diagnosis and management due to the complex anatomy and the necessity to restore both bony and soft tissue integrity. The treatment approach for floating clavicle injuries can vary depending on the severity and displacement of the fractures or low-demand patient. Conservative management with immobilization may be suitable for non-displaced fractures, while surgical intervention is often necessary for displaced or unstable fracture-dislocation or young active patients to achieve anatomical reduction and stable fixation.

In this case report, we present the details of a patient with a floating clavicle injury following a high-energy trauma. We discuss the clinical presentation, radiographic findings, surgical management, and postoperative outcomes. Furthermore, we provide an updated review of the literature on floating clavicle injury, discussing various mechanisms of injury, treatment options, and outcomes. Our objective is to contribute to the existing body of knowledge on this rare injury and offer insights into the optimal management strategies for similar cases.

The patient consented to the publication of this case report. The paper was approved by the ethics committee of our institute.

## Case presentation

**Figure 1:**
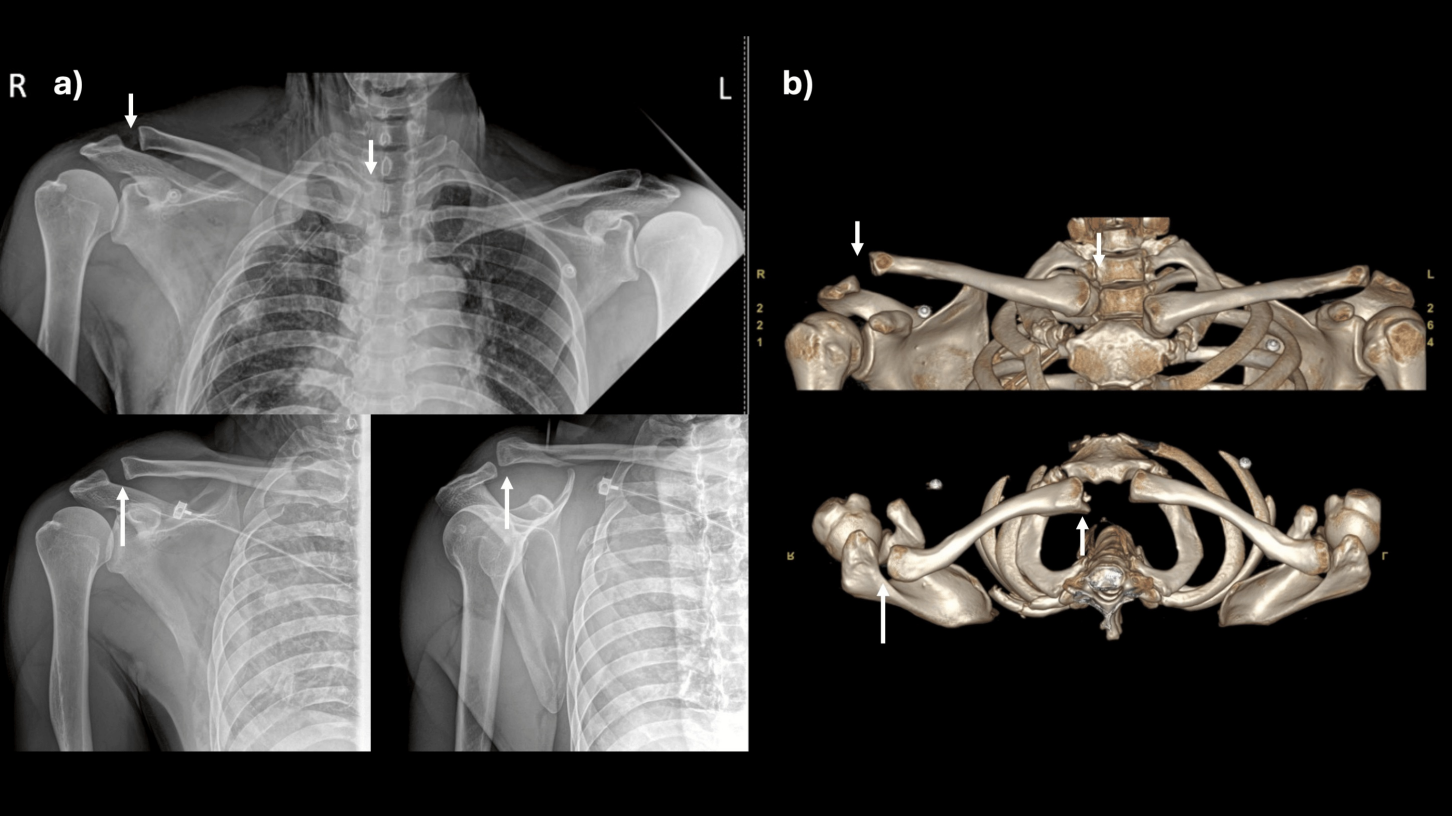
Radiographic examination of the injury. (a) The radiographs of both clavicles, right clavicle anteroposterior view, and transcapular view. (b) The 3D CT scan demonstrated superior displacement of the acromioclavicular joint (arrow) and posterosuperior displacement of the sternoclavicular joint (arrow).

A 43-year-old male presented to the emergency department following a motorcycle accident with multiple injuries, including right shoulder pain. He was evaluated and resuscitated following the Advanced Trauma Life Support (ATLS) protocol. Associated injuries included a subdural hemorrhage, right-sided fractures of the 2nd to 6th ribs with hemothorax, and an anterior orbital wall fracture. Once the patient’s condition was stabilized, a physical examination revealed tenderness and deformity over the acromioclavicular (AC) and sternoclavicular (SC) joints. The range of motion was limited due to pain, but the distal neurovascular status was intact. Radiographs of both clavicles demonstrated widening of the AC joint with superior displacement of the SC joint (Figure 1A). A 3D computed tomography scan confirmed these findings, showing superior displacement of the AC joint (Rockwood type V) and posterosuperior displacement of the SC joint (Figure 1B). After discussing treatment options, the patient, who was active and had high functional demands, opted for surgical stabilization.

Surgical technique

After administering general anesthesia, the patient was positioned in the semi-Fowler position with back support provided by a pillow to achieve a 30-degree elevation of the upper body. The patient's arm was left free to allow dynamic examination of the SC joint. Anesthesia examination included assessing the degree and direction of instability, reducibility, and hyperlaxity. The patient's arm was then placed freely beside the body.

Open stabilization of the sternoclavicular joint with FiberTape (ligament bracing)

**Figure 2:**
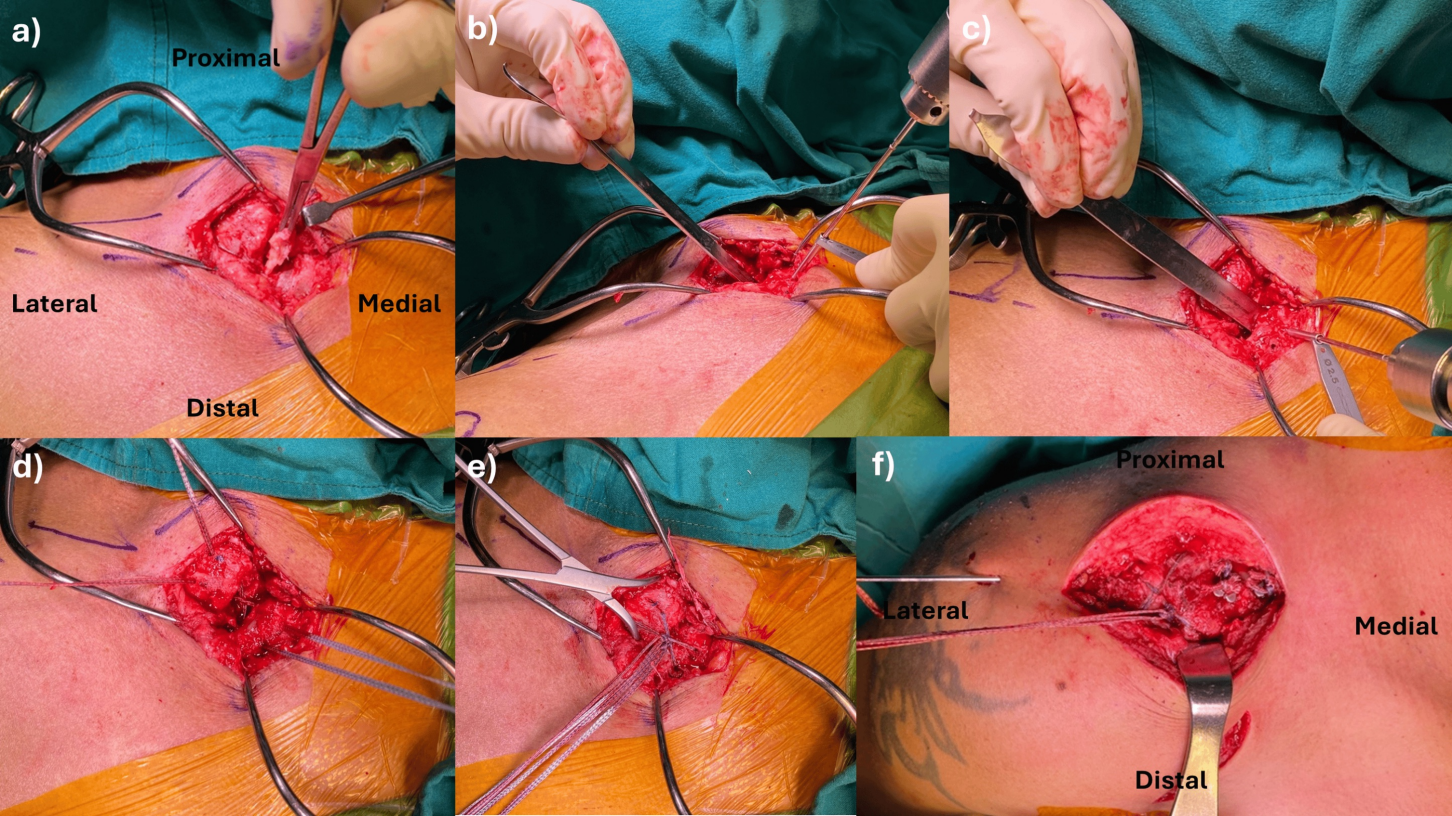
Surgical technique for SC and AC joint stabilization. (a) The torn meniscal disc is resected after exposing the SC joint. (b and c) The bone tunnels were created at a 45-degree angle with a metal malleable retractor to protect the posterior sternal structures. (d and e) A figure-of-eight suture was tied over the anterior SC joint area. (f) The figure-of-eight suture with a suture box stabilized the AC joint. AC: acromioclavicular; SC: sternoclavicular.

**Figure 3:**
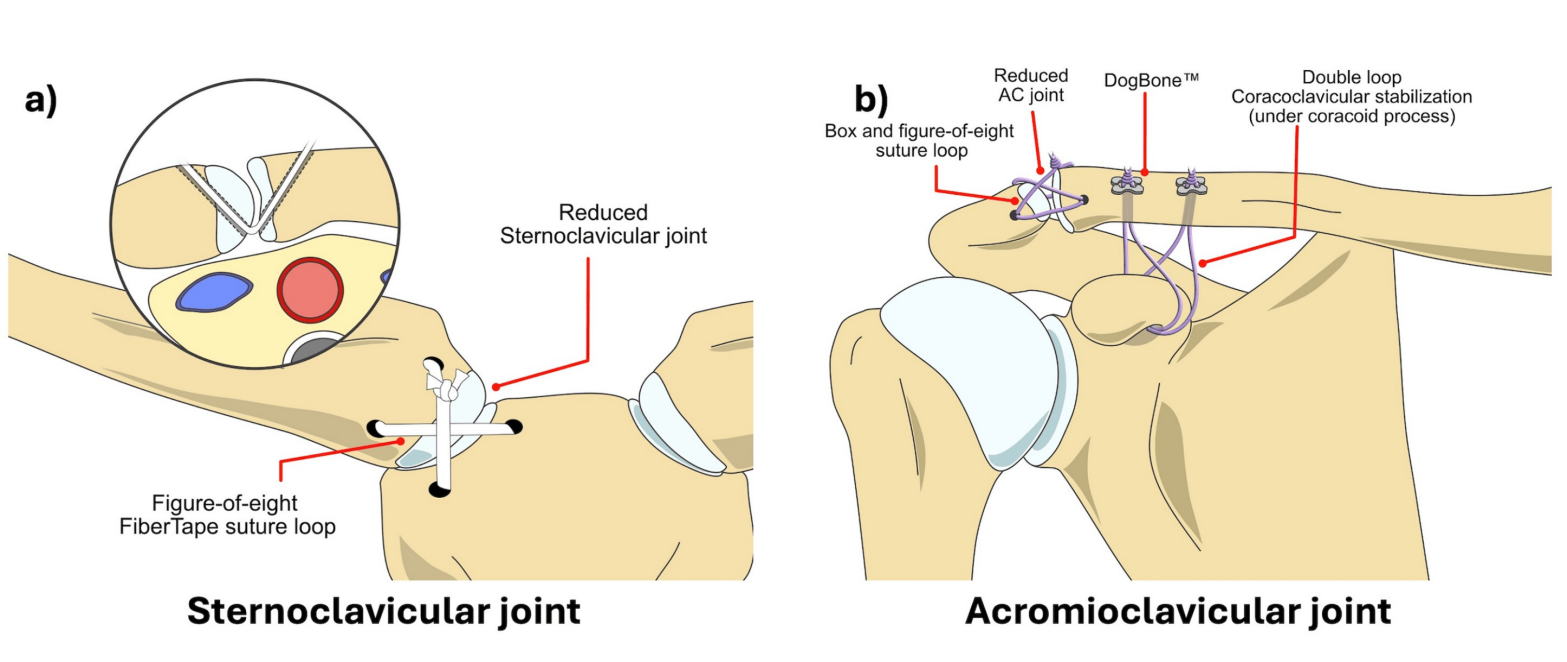
The illustration demonstrating the stabilization of (a) the SC and (b) AC joints. AC: acromioclavicular; SC: sternoclavicular. The figure is the original work of the authors.

A skin incision approximately 5 cm long was made from the medial clavicle to the sternum. The SC joint capsule was opened using electrocautery and mobilized with a periosteum elevator. The platysma was identified and mobilized and will be reconstructed later. Releasing the anterior joint capsule is necessary to gain good access to the medial clavicle and sternum. The sternum and medial clavicle were mobilized until a metal retractor could be placed as a drill protector on the posterior cortical bone of the sternum and clavicle. The torn disc was then resected (Figure 2A). After clearly identifying the medial end of the clavicle and sternum, two oblique holes of 2.5 mm diameter were drilled at each end, positioned at least 1 cm away from the edge to prevent bone cut through. As the holes were drilled from anterior to posterior, a metal protector was inserted at the posterior cortex of the clavicle and sternum to safeguard vital organs (Figures 2B, 2C). An 18-gauge needle was used to pass a polydioxanone (PDS) suture, acting as a shuttle suture. Subsequently, two fiber tapes were fed through the drill channels in a figure-of-eight configuration (Figures 2D, 2E). The joint was repositioned, and the two fiber tapes were knotted anteriorly (Figure 3A). Dynamic stability was tested under direct visualization. The capsule and platysma were meticulously reconstructed. Finally, subcutaneous and skin closure was performed.

Open coracoclavicular and acromioclavicular stabilization

We used a dual incision technique for this procedure. A 5-cm vertical incision was made at the tip of the coracoid, and the deltoid fibers were split to expose the tip, base of the coracoid, and the coracoacromial (CA) ligament. Using a suture retriever, two strands of No. 5 FiberWire (Arthrex, Naples, FL) and two strands of FiberTape® (Arthrex) were passed around the base of the coracoid from medial to lateral, ensuring all sutures remained behind the CA ligament and proximal to the coracoid tip. A second 5-cm horizontal incision was made over the AC joint and distal clavicle, where the deltotrapezial fascia was split to expose and preserve the AC ligament. A 2.5-mm hole was drilled 2.5 cm medial to the AC joint, and a second tunnel was created 1.5 cm medial to the first. Using a shuttle relay technique, one strand each of FiberWire and FiberTape® was sequentially drawn through each clavicular tunnel. After achieving the anatomical reduction of the AC joint, a temporary 1.8 mm K-wire was inserted through the AC joint, and a lateral loop was securely tied over the Dog Bone™ Button (Arthrex) using a non-sliding knot technique. Intraoperative fluoroscopy confirmed successful reduction, followed by tying the medial loop over the Dog Bone™ Button. The AC joint meniscus was identified and excised. Two tunnels were drilled anterior-to-posterior, one crossing the lateral clavicle edge and the other crossing the anterior acromion. A single strand of FiberTape® was passed through the distal and acromial tunnels in a figure-of-eight configuration, securely tied around the AC joint, incorporating capsular remnants if feasible (Figure 2F and Figure 3B). Dynamic joint stability was tested under direct visualization, with arm movement through the shoulder girdle range. Once hemostasis was ensured, the deltotrapezial fascia was closed and plicated over the AC joint capsule. If possible, the platysma was repaired, followed by the closure of the skin in layers and the insertion of a Redivac drain.

**Figure 4:**
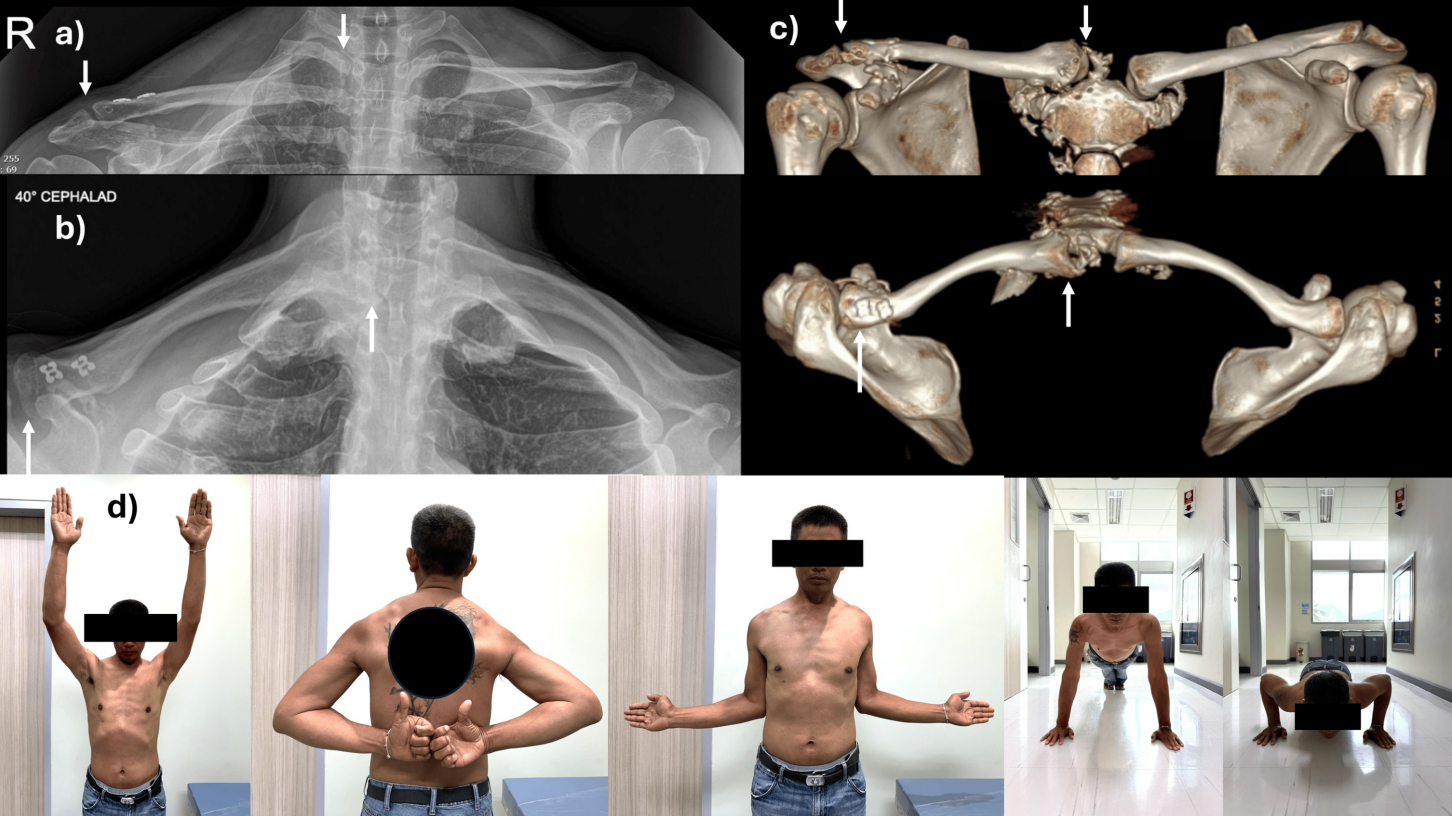
Postoperative radiographic and clinical examination of the patient. (a and b) Radiographs of both clavicles in the anteroposterior and cephalad tilt views. (c) 3D CT scan of both clavicles. (d) The clinical function of the patient at one-year follow-up. The arrow showed reduced acromioclavicular and sternoclavicular joints.

The patient was immobilized with an arm sling for four weeks, and the trans-AC joint K-wire was removed at that time. Passive range of motion exercises up to shoulder level were initiated once postoperative pain subsided. Full passive motion as tolerated was permitted after four weeks, and active motion began after six weeks. At the three-month follow-up, the patient had regained a full range of motion without pain. By the 12-month follow-up, the patient had returned to full function with previous work activities, reported no pain with a visual analog scale score of 0, had a DASH (Disabilities of the Arm, Shoulder, and Hand) score of 2.7, and a Constant-Murley Score of 95. Radiographs showed a near anatomical reduction of the AC and SC joints. The patient expressed satisfaction with the surgical outcome (Figure 4).

## Discussion

Traumatic floating clavicle, also known as bipolar dislocation or panclavicular dislocation, is an exceptionally rare injury characterized by simultaneous dislocation of the SC and AC joints. In the literature, these terms are often used interchangeably to describe this unique injury pattern, where the clavicle becomes "floating" due to the loss of its attachment at both ends. The complexity of this condition lies in the unique disruption of the shoulder girdle’s continuity, which can lead to significant instability and functional impairment. This case report highlights a successful surgical intervention for a 43-year-old male who sustained this injury in a high-energy motorcycle accident, and it provides an updated review of the literature regarding this unusual condition.

Floating clavicle injuries are rare, accounting for a small percentage of shoulder girdle injuries. To understand the clinical characteristics of these injuries, case reports of floating clavicle injuries were searched in the PubMed database from 2000 to 2024, and 58 cases were found. The injury characteristics, mechanisms of injury, treatment options, and reported outcomes were recorded. The mean age was 45.4 years, ranging from 13 to 82 years old. These injuries are typically caused by high-energy trauma such as motor vehicle accidents, falls from significant heights, or direct blows to the shoulder region. The incidence of these injuries resulting from high-energy mechanisms is 89.5%, though they can also occur following low-energy trauma such as slip and fall (10.5%). High-energy mechanisms often involve associated chest injuries, including the fracture of ribs or hemopneumothorax, which are found in 41.2% of cases. The characteristics of the floating clavicle injuries vary: pure dislocation of both the AC and SC joints occurs in 46.6% of cases, dislocations with fractures at the other ends in 27.6%, and fractures in both ends in 25.9%. On the medial side, the majority of injuries are dislocation (58.6%), followed by fracture (29.3%) and fracture-dislocation (12.1%), with anterior (70.3%) and posterior displacement (13.5%) of the injury. On the lateral side, the occurrence is nearly equal between dislocation (50%) and fracture (48.3%) with posterior (50%) and superior displacement (39.3%) of the injury.

Diagnosis can be challenging due to the complex anatomy involved and the need for precise imaging to confirm the extent of the injury. Previous studies have reported instances of missed or delayed diagnoses [3,10], which may underestimate the incidence of this injury. A high index of suspicion in polytrauma patients and careful examination of the entire clavicle is advised to avoid missing a bipolar injury. A full-length clavicle radiograph is recommended [11], and the CT scan is accepted as the gold standard for evaluating this complex deformity [1,12]. In this case, initial radiographs suggested a superior displacement of the AC joint and a suspected dislocation of the SC joint, which was subsequently confirmed by a 3D CT scan.

Two main hypotheses have been proposed to explain the mechanism of injury. The simultaneous theory suggests that elastic potential energy results in both dislocations happening at the same time. The consecutive theory, on the other hand, suggests that an anterior impact on the lateral end of the clavicle first causes the SC joint to dislocate, followed by the dislocation of the AC joint [1,9].

Management of floating clavicle injuries varies depending on the patient's activity level, the extent of displacement, and associated injuries. In the current review, most of the studies suggested surgical treatment (84.5%) over conservative treatment (15.5%). While conservative treatment [13,14] may be appropriate for non-displaced or minimally displaced injuries in low-demand patients, surgical intervention is often indicated for active individuals or those with significant displacement and instability. The goal of surgical treatment is to achieve anatomical reduction and stable fixation to restore both bony and soft tissue integrity.

Sanders et al. [15] reported on six patients with floating clavicle injuries treated conservatively. Four of these patients experienced persisting symptoms such as pain and limited ROM, necessitating later surgical reconstruction of their AC joint. Similarly, Lee et al. [9] reported a series of 11 patients with these injuries. Although all patients were satisfied with the results, those with unreduced dislocation or fracture at one or both ends of the clavicle had lower functional scores, deformity, or residual pain.

There is no consensus on the surgical treatment for floating clavicles due to their rarity. The rationale is that when there is a dislocation or fracture at each end of the clavicle, each lesion should be managed according to the specific classification and grade as if it were an isolated injury. Unstable and displaced fractures or dislocations, particularly Rockwood type IV, V, and VI, and Neer type II and V injuries, should be managed surgically.

Various surgical techniques and modes of fixation of AC joint and SC joint dislocation have been proposed, including Kirschner wire [1], tension band wiring [1,16,17], cerclage wire [17], compression screw [18], T-plate [16,19,20], anatomical locking plate [10,11,21-27], and hook plate [7,9,10,12,23,24,28-31]. Additionally, ligamentous reconstruction using polyester fiber tape [32], Mersilene tape [5], polyester mesh [33], and tendon graft [3,8,22,34,35] have been utilized. Claviculectomy has been reported in a case with delayed treatment for four years, resulting in a good result [36]. While no single method has been proven superior for all cases, the choice of technique should be tailored to the specific injury pattern, patient factors, and surgeon expertise. K-wire or tension band wiring are simple and relatively inexpensive options, but their major disadvantages were hardware-related problems, risk of migration, and limited stability compared to other methods. Miniplate or anatomical plate fixation achieves excellent stability from the locking mechanism; however, it requires extensive dissection, may cause irritation necessitating implant removal, and carries the risk of stress shielding or peri-implant fracture. One reported complication is a peri-implant fracture at the midshaft clavicle, which can occur due to stress riser in that area when both ends are fixed with plates [23]. Hook plates are effective in AC joint injury and can also be used for SC joint injury. However, they require implant removal and pose a risk of injury to surrounding tissues, particularly the retromediastinal structure in SC joint injury. Ligamentous reconstruction aims to restore the natural biomechanics of the joint by reconstructing the ligamentous structures, but it is typically more complex, requires a high level of surgical skill, and is relatively expensive when using allograft. Understanding the pros and cons of each approach helps in making informed decisions that align with the goals of achieving stable fixation, promoting healing, and minimizing complications.

The sequence of surgical procedures for these injuries remains controversial. Schemitsch et al. [12] recommended fixing the more severely affected side first. Feng et al. [24] supported this strategy, finding that once the more severely affected side was stabilized, the other end could be passively reduced. Thyagarajan et al. [33] fixed the SC joint first in their report and found that the AC joint (Rockwood type III) was passively reduced. Conversely, Lee et al. [9] managed the AC injury with a hook plate first and then performed open reduction of the SC joint after a failed closed reduction. In our case, we opted to address the SC joint injury first. Although the radiograph revealed comparable severity of displacement on both sides, our rationale was to first connect the clavicle to the axial skeleton. With stable proximal fixation, we could then restore the distal part, achieving stable and anatomical reconstruction.

In this case, the surgical approach involved open stabilization of the SC joint using FiberTape sutures and stabilization of the AC joint using Dog Bone™ Button and suture cerclage techniques. The use of FiberTape for SC joint stabilization provides robust fixation, allowing for early mobilization and reducing the risk of recurrent dislocation. Similarly, the combination of Dog Bone™ Button and suture cerclage for AC joint stabilization ensures secure fixation and supports the restoration of joint function. The patient demonstrated excellent recovery, regaining nearly full range of motion without pain at the three-month follow-up. At the 12-month follow-up, the patient returned to full functional activities, reported no pain, and achieved a visual analog scale score of 0, a DASH score of 2.7, and a Constant-Murley Score of 95, which are comparable to the previous studies [9,24,28]. Radiographs showed a near anatomical reduction of both the AC and SC joints and the patient expressed satisfaction with the surgical outcome (Tables 1, 2).

**Table 1:** Case-by-case floating clavicle injury characteristics during 2000-2024. * The hook plate was removed approximately six months after the operation. SCJ: sternoclavicular joint; ACJ: acromioclavicular joint; TBW: tension band wiring; CC: coracoclavicular; ROM: range of motion; IR: internal rotation; ER: external rotation; LCP: locking compression plate; CMS: Constant-Murley Score; DASH: Disabilities of Arm, Shoulder, and Hand; VAS: visual analog scale; ASES: American Shoulder and Elbow Surgeons Score; NA: not applicable; PDS: polydioxanone; FF: forward flexion; Sat: satisfaction score; SC: sternoclavicular.

Authors (year)	Age	Cause of injury	Characteristic of the injury		Associated injury	Time to surgery	Treatment		Follow-up and reported outcome	Complication
			Medial side	Lateral side			Medial side	Lateral side		
Scapinelli (2004) [1]	18	High-speed automobile accident	Anterosuperior SCJ dislocation	Posterior ACJ dislocation (Rockwood IV)	Fracture of contralateral scapular body, Pneumothorax	19 days	Transarticular K-wires	TBW	2 years: full ROM, normal motor power	Broken wire of SCJ fixation
Schemitsch et al. (2011) [12]	42	Motorcycle accident	Anterior SCJ dislocation	Posterior ACJ dislocation (Rockwood IV)		8 months	Hook plate	Hook plate	1 year: flexion 170, ER 45, IR L5	Hardware removal due to limited motion
	49	Car accident	Anterior SCJ dislocation	Posterior ACJ dislocation (Rockwood IV)	Ribs fracture, T-spine fracture, Iliac crest fracture	Acute	Conservative	Hook plate	8 months: flexion 160, normal rotation	
Serra et al. (2011) [2]	71	Falling from stairs	Anterior SCJ fracture-dislocation	Distal clavicle fracture (Neer I)		NA	Conservative	Conservative	6 months: abduction 160, flexion 160, extension 35, adduction 25, ER 50, IR 90	
Choo et al. (2012) [5]	48	Traffic accident	Anterior SCJ dislocation	Superior ACJ dislocation (Rockwood V)	Ipsilateral forearm and wrist injury		Mersilene tape (sternum, medial clavicle, and 1^st^rib)	Hook plate and cc-stabilization with Mersilene tape	5 months: full ROM	
Jiang et al. (2012) [16]	41	Car accident	Anterosuperior SCJ dislocation	Posterior ACJ dislocation (Rockwood IV)	Multiple ribs fractures, hemothorax	Acute	T-plate	TBW	2 years: abduction 150, flexion 160, extension 60, normal rotation	Hardware removal at 6 months
Madhuri et al. (2012) [13]	13	Hit by sport utility vehicle	Anterosuperior SCJ physeal injury	Posteroinferior ACJ physeal injury	Sensory deficit on the medial aspect of the forearm	Acute	Conservative	Conservative	18 months: full ROM, remodeling with joint congruity	
Schuh et al. (2012) [17]	23	Motorcycle accident	Anterior SCJ dislocation	Posterior ACJ dislocation (Rockwood IV)	Lung contusion, multiple rib fractures, sacral fracture	21 days	Wire cerclage	TBW	18 months: full ROM, normal activity	Hardware removal at 8 weeks
Yurdakul et al. (2012) [18]	21	Car accident	Anterosuperior SCJ dislocation	Posteroinferior ACJ dislocation	Scapular body fracture	21 days	Compression screw	Compression screw	3 months after hardware removal: clinically stable, good ROM, flexion 160, abduction 110	Hardware removal
Gouse et al. (2013) [4]	19	Motorbike accident	Anterior SCJ dislocation	Distal clavicle fracture		NA	Conservative	Conservative	18 months: mild deformity, no functional disability, full ROM	
Schliemann et al. (2014) [19]	31	Bicycle accident	Anterior SCJ fracture-dislocation	Distal clavicle fracture (Neer IIb)	Posterior shoulder dislocation with Hill-Sachs	5 days	Figure of eight PDS cerclage	T plate 2.4 mm	6 months: full ROM	Plate removal at 1 year due to implant irritation
Thyagarajan et al. (2015) [33]	51	Car accident	Posterior SCJ dislocation	ACJ dislocation (Rockwood III)		21 days	Lockdown to 1^st^ rib (polyester surgical mesh device)	Lockdown (Polyester surgical mesh device)	6 months: full ROM. 14 months: Constant score 96, Oxford score 46, Nottingham score 90	
Sopu et al. (2015) [20]	52	Fall from bicycle	Anterior SCJ fracture- dislocation	Distal clavicle fracture	Metacarpal fracture	Acute	Small T plate	Conservative	5 months: no pain, no functional deficit	
Talboys (2016) [14]	79	Falling	Medial clavicle fracture (Allman III)	Distal clavicle fracture (Neer I)		NA	Conservative	Conservative	3 months: pain-free, no functional deficit	
Yalizis et al. (2016) [10]	38	Bicycle accident	Medial clavicle fracture	Distal clavicle fracture (Neer II)	3^rd^ 4^th^ ribs fracture	Lateral - 4 days; medial - 43 days	Distal clavicle LCP	Hook plate	Remove hook plate, 3 months - full ROM	Missed medial side injury
Ogawa et al. (2017) [21]	74	Hit by the side view mirror of a moving car	Extraarticular medial clavicle fracture (Robinson 1B1) (anterior)	Intraarticular lateral clavicle fracture (Robinson 3B2) (posterior)		7 days	Conservative	Distal clavicle LCP	3 years: DASH 5.0, Oxford score 47, ASES 91.6, flexion 165, abduction 80	Implant removal at 1 year due to irritation
Okano et al. (2017) [7]	45	Fell from a ladder	Anterior SCJ dislocation	ACJ dislocation (Rockwood III)	Hemothorax, 7^th^rib fracture	10 days	Conservative	Modified Cadenat’s	12 months: mild discomfort around ACJ, slight anterior protrusion of SCJ, full ROM	
	36	Compress with machine	Posterior SCJ dislocation	ACJ dislocation (Rockwood III)	Depressed skull fracture, epidural hematoma, Coracoid fracture, hemothorax, scapular body fracture	1 day	FiberWire suture	Hook plate	12 months: full ROM, no residual symptoms	
Prasetia et al. (2017) [22]	32	Motorcycle accident	Anterior SCJ dislocation	Superior ACJ dislocation (Rockwood V)	Coracoid fracture, clavicle fracture, multiple ribs fracture	24 days	Figure-of-eight semitendinosus autograft	Clavicular plating, coracoid screw, Semitendinosus autograft reconstruction	6 months: no pain, ASES score = 84, full ROM	
Lee et al. (2018) [9]	49	Falling	Anterior SCJ dislocation	ACJ dislocation (Rockwood V)	Rib fracture, pneumothorax	8 weeks	Neglect	Hook plate	28 months: CMS = 92, flexion = 160, ER = 60, abduction = 130, IR = T12 level	*
	59	Motorcycle accident	SCJ dislocation	ACJ dislocation	Rib fracture, hemothorax, pneumothorax	NA	Neglect	Closed reduction	10 months: CMS = 85	
	41	Falling	SCJ dislocation	ACJ dislocation	Rib fracture, pneumothorax	2 weeks	Closed reduction	Hook plate	12 months: CMS = 92	*
	40	Traffic accident	SCJ dislocation	Fracture	Rib fracture, hemothorax, pneumothorax	4 days	Closed reduction	Hook plate	13 months: CMS = 96	*
	45	Pedestrian traffic accident	SCJ dislocation	Fracture	Rib fracture, pneumothorax, liver laceration		Closed reduction	Closed reduction (CC widening)	11 months: CMS = 85, flexion = 160, ER = 60, abduction = 140, IR = T12 level	
	45	Slip	SCJ dislocation	Fracture		3 days	Open ligament repair	Hook plate	10 months: CMS = 94	*
	35	Bicycle injury	SCJ dislocation	Fracture		4 days	Closed reduction	Hook plate	9 months: CMS = 96	*
	36	Slip	SCJ dislocation	Fracture		3 days	Closed reduction	Hook plate	16 months: CMS = 96	*
	54	Traffic accident	SCJ dislocation	Fracture	Peritoneal hemorrhage		Neglect	Closed reduction (AC, CC widening)	26 months: CMS = 72	
	50	Fall	Fracture	ACJ dislocation (Rockwood III)	Rib fracture, T12 fracture, hemothorax		Closed reduction	Closed reduction (CC widening)	10 months: CMS = 83, flexion = 150, ER = 50, abduction = 130, IR = T12 level	
	34	Slip	Fracture	Fracture			Closed reduction	Closed reduction	8 months: CMS = 96	
Dev et al. (2020) [34]	37	Bicycle accident	Anterior SCJ fracture-dislocation	Posterior ACJ dislocation (Rockwood IV)		4 months	Figure-of-eight semitendinosus allograft	Tightrope (Arthrex)	6 months: minimal pain and some stiffness with abduction	
Salmas et al. (2020) [3]	65	Motorcycle accident	Posterosuperior SCJ dislocation	Posterior ACJ dislocation (Rockwood IV)		6 months	Figure-of-eight semitendinosus autograft	CC-stabilization	1 year: pain-free, full ROM	
Zou et al. (2020) [23]	58	Hitting by an object falling from a height	Anterior SCJ dislocation	ACJ dislocation (Rockwood V)		Medial - acute; lateral - 1 month	Distal clavicle LCP	Hook plate	11 months: full ROM	Missed ACJ injury. Midshaft clavicle fracture (peri-implant)
De Ruiter et al. (2021) [11]	23	High-speed motor vehicle accident	Medial clavicle fracture	Lateral clavicle fracture	4-6^th^ cervical spine fracture, left vertebral artery dissection, transverse process fracture, 5-8^th^ thoracic spine, 1^st^-2^nd ^ribs fracture, lung contusion	Acute	2.7/3.5 LCP	Distal clavicle LCP	6 weeks: abduction = 65, adduction = 20	
Mesregah et al. (2021) [36]	26	High-velocity motor vehicle accident	Anterosuperior SCJ dislocation	Posteroinferior ACJ dislocation		4 years	Claviculectomy	Claviculectomy	1 year: full ROM, normal activity	
Moreno-Fenoll et al. (2021) [25]	51	High-velocity biking accident	Medial clavicle fracture	Posterior ACJ dislocation (Rockwood IV)	Concomitant ribs fracture, pulmonary contusion, pleural effusion	5 days	Distal ulnar plate	MINAR® implant	10 months: full ROM, pain-free, satisfy	
Sono et al. (2021) [26]	82	Falling	Midshaft clavicle fracture	Distal clavicle fracture		2 days	Anterior plate	Superior plate	4 months: union, no complication	
Bansal et al. (2022) [8]	22	Fall from stairs	Anterior SCJ dislocation	Posterior ACJ dislocation (Rockwood IV)			FiberWire	Gracilis graft/FiberTape coracoclavicular stabilization	1 year: near normal ROM	
Jacob et al. (2022) [32]	62	High-velocity biking accident	Anterosuperior SCJ dislocation	Posterior ACJ dislocation (Rockwood IV)		20 days	Figure of eight with FiberTape + cerclage with Vicryl 1-0	Horizontal cerclage with FiberTape	6 months: pain-free, 160 passive and 140 active anteversion	
Liang et al. (2022) [30]	56	Falling from bicycle	Medial clavicle fracture (anterior displace)	Distal clavicle fracture (superior)		4 days	Contoured T plate + K-wire	Hook plate	1 year: full ROM flexion = 150	Plate removal due to irritation
Xing et al. (2022) [31]	76	Traffic injury	Medial clavicle fracture (Robinson 1B2)	Distal clavicle fracture (Robinson 3B2)		7 days	Trans-sternoclavicular LCP	Hook plate	3 months: DASH = 40	
Oladeji et al. (2023) [35]	NA	NA	Locked posterior SCJ dislocation	ACJ dislocation (Rockwood III)			Figure-of-8 gracilis allograft and nonabsorbable suture reconstruction	Semitendinosus allograft and nonabsorbable suture	NA	
Yu et al. (2023) [29]	26	Motorcycle accident	Medial shaft clavicle with anterior SCJ dislocation	Distal clavicle fracture		3 days	Reverse distal clavicle LCP + Suture Across sternum	Hook plate	13 months: upper limb function score = 2.27	
Feng et al. (2023) [24]	54	Fall from height	Anterior SCJ dislocation	Posterior ACJ dislocation (Rockwood IV)		4 days	Hook plate	Conservative	13 months: FF = 150, DASH = 18.3, CMS = 72, VAS = 4	
	26	Crashing	Anterior SCJ fracture-dislocation	Posterior ACJ dislocation (Rockwood IV)	Tooth fracture	8 days	Hook plate	Conservative	14 months: FF = 165, DASH = 3.3, CMS = 96, Sat = 10, VAS = 0	Remove implant from irritation
	58	Car accident	Anterior SCJ dislocation	Fracture	Chest/brain injury	147 days	Hook plate	LCP	37 months: FF = 160, DASH = 10, CMS = 90, Sat = 9, VAS = 1	
	47	Motorcycle accident	Anterior SCJ dislocation	Fracture-dislocation	None	5 days	Conservative	Hook plate	15 months: FF = 165, DASH = 6.7, CMS = 94, Sat = 10, VAS = 0	
	62	Car accident	Medial clavicle fracture (Robinson 1B2)	Distal clavicle fracture (Neer III)	Chest, ipsilateral scapular	32 days	Revision hook plate	Hook plate	14 months: FF = 150, DASH = 11.7, CMS = 88, Sat = 9, VAS = 2	
	29	Fall from height	Anterior SCJ fracture-dislocation (Robinson 1B2)	III dislocation	Chest, ipsilateral scapular	12 days	Hook plate	Hook plate	31 months: FF = 155, DASH = 6.7, CMS = 90, Sat = 9, VAS = 0	Remove implant from irritation
	64	Fall from height	Medial clavicle fracture (Robinson 1B1)	Distal clavicle fracture (Neer II)	Ipsilateral scapular	10 days	LCP	Conservative	73 months: FF = 170, DASH = 5, CMS = 92, Sat = 10, VAS = 0	
Zhang et al. (2023) [28]	34	4 car accidents, 1 fall from height, 1 fall from motorcycle, 1 slip and fall	Fracture	Fracture	2 cases of combined rib fractures, 1 femur fracture, 1 head injury		New SC hook plate	Hook plate	ASES = 98, DASH = 1.7, VAS = 0, CMS = 99	
	56		Fracture	Dislocation			New SC hook plate	Hook plate	ASES = 92.5, DASH = 5.8, VAS = 1, CMS = 94	
	68		Fracture	Fracture			New SC hook plate	Hook plate	ASES = 90, DASH = 6.7, VAS = 2, CMS = 89	
	49		Anterior SCJ dislocation	Dislocation			New SC hook plate	Hook plate	ASES = 96, DASH = 2.5, VAS = 0, CMS = 95	
	40		Anterior SCJ dislocation	Fracture			New SC hook plate	Hook plate	ASES = 96, DASH = 3.3, VAS = 0, CMS = 94	
	51		Anterior SCJ dislocation	Dislocation			New SC hook plate	Hook plate	ASES = 95, DASH = 2.5, VAS = 1, CMS = 96	
	60		Posterior SCJ dislocation	Fracture			New SC hook plate	Hook plate	ASES = 93, DASH = 5, VAS = 1, CMS = 92	
Timilsina et al. (2024) [27]	35	Road traffic accident	Medial clavicle fracture (first diagnosed as anterior SCJ dislocation)	Distal clavicle fracture (Neer IIB)	None	Acute NA	3.5 mm LCP	Recon plate	14 months: full ROM, DASH = 0	

**Table 2:** Summary of the characteristics of the cases during 2000-2024.

Characteristic	No. (%)
Mean age (years)	45.4
Mechanism of injury (N = 57)	
Car accident	16 (28.1%)
Motorcycle accident	12 (21.1%)
Bicycle accident	7 (12.3%)
Pedestrian	3 (5.3%)
Fall from height	10 (17.5%)
Slip and fall	6 (10.5%)
Crashing	1 (1.8%)
Hitting by an object	1 (1.8%)
Compress with machine	1 (1.8%)
Degree of mechanism of injury	
High energy	51 (89.5%)
Low energy	6 (10.5%)
Associated chest injury (N = 51)	21 (41.2%)
Characteristic of sternoclavicular joint injury	
Dislocation	34 (58.6%)
Fracture	17 (29.3%)
Fracture-dislocation	7 (12.1%)
Characteristic of acromioclavicular joint injury	
Dislocation	29 (50%)
Fracture	28 (48.3%)
Fracture-dislocation	1 (1.7%)
Characteristic of both side injuries (medial/lateral)	
Dislocation/dislocation	27 (46.6%)
Fracture/dislocation	3 (5.2%)
Dislocation/fracture	13 (22.4%)
Fracture/fracture	15 (25.9%)
Treatment	
Conservative	9 (15.5%)
Surgery	49 (84.5%)

## Conclusions

This case underscores the importance of a tailored surgical approach in managing complex shoulder girdle injuries such as the traumatic floating clavicle. The literature supports the efficacy of surgical stabilization in active patients, highlighting the need for individualized treatment plans based on the severity of the injury and the patient’s functional demands. Further studies are needed to establish standardized treatment protocols and long-term outcomes for this rare but challenging condition.
